# Role of Ginseng and L-Carnitine in Modulating Exercise Endurance and Oxidative Stress in Rats

**DOI:** 10.3390/nu17030568

**Published:** 2025-02-03

**Authors:** Kakanang Posridee, Sajeera Kupittayanant, Pornthep Rachnavy, Anant Oonsivilai, Ratchadaporn Oonsivilai

**Affiliations:** 1 Health and Wellness Research Unit, School of Food Technology, Institute of Agricultural Technology, Suranaree University of Technology, Nakhon Ratchasima 30000, Thailand; posridee.ka@gmail.com; 2 School of Preclinical Sciences, Institute of Science, Suranaree University of Technology, Nakhon Ratchasima 30000, Thailand; sajeera@sut.ac.th; 3 School of Sport Science, Institute of Science, Suranaree University of Technology, Nakhon Ratchasima 30000, Thailand; rachnavy@sut.ac.th; 4 School of Electrical Engineering, Institute of Engineering, Suranaree University of Technology, Nakhon Ratchasima 30000, Thailand

**Keywords:** ginseng extract, L-carnitine, exercise endurance, muscle, liver, glycogen, oxidative stress, AMPK, *PGC-1α*, mitochondrial biogenesis

## Abstract

Ginseng and L-carnitine are natural compounds often used as dietary supplements to enhance athletic performance. However, their combined effects on exercise endurance remain unclear. **Objectives**: This study aimed to investigate the effects of ginseng extract and L-carnitine supplementation on exercise endurance in a rat model. **Methods**: Male Wistar rats were divided into 10 groups (*n* = 5 per group): control, ginseng extract (250 and 500 mg/kg/day), L-carnitine (250 and 500 mg/kg/day), and combined treatment. Half of the groups underwent a 16-day exercise training program of swimming without loading. Exercise endurance was assessed using a tail-suspended forced swimming test. Relative organ weight, glycogen content, blood biochemistry, and gene expression were analyzed. **Results**: Both ginseng extract and L-carnitine supplementation significantly increased exercise endurance, particularly in the exercise group. Ginseng extract and L-carnitine also increased liver glycogen content and upregulated the expression of *AMPKα1* and *PGC-1α* genes in the liver and muscle. In addition, both supplements reduced oxidative stress by decreasing MDA levels and increasing SOD activity. **Conclusions**: Ginseng extract and L-carnitine supplementation may enhance exercise endurance by improving energy metabolism, reducing oxidative stress, and upregulating key genes involved in mitochondrial biogenesis.

## 1. Introduction

Exercise performance is influenced by various physiological, psychological, and nutritional factors. Recently, the use of dietary supplements to enhance athletic performance has gained considerable interest. Ginseng and L-carnitine are two supplements investigated for their potential ergogenic effects. Ginseng is a herbal medicine that has been used for centuries to improve physical and mental health. Several studies have examined the ergogenic effects of ginseng on athletic performance. Park et al. [1] and Chen et al. [2] found that ginseng supplementation increases endurance, reduces fatigue, and improves recovery times in athletes. The mechanisms underlying these effects are complex and may involve multiple factors, including increased energy production, reduced oxidative stress, and improved muscle function. L-Carnitine is a naturally occurring compound that plays a crucial role in fatty acid oxidation. It transports long-chain fatty acids into mitochondria, where these fatty acids are oxidized to produce energy. L-carnitine supplementation can positively affect exercise performance, particularly endurance-based activities [3]. The mechanisms underlying the ergogenic effects of L-carnitine are related to its role in fatty acid metabolism and its potential to reduce muscle fatigue.

Exercise induces oxidative stress, leading to muscle damage and fatigue. Antioxidants such as those found in ginseng and other natural sources protect against oxidative stress and improve exercise performance. Previous research has highlighted the role of oxidative stress in exercise-induced muscle damage and the potential benefits of antioxidant supplementation [4,5]. Key regulators of energy metabolism and exercise adaptation include AMP-activated protein kinase (*AMPK*) and peroxisome proliferator-activated receptor-γ coactivator-1α (*PGC-1α*). *AMPK* activation stimulates glucose uptake, fatty acid oxidation, and mitochondrial biogenesis, while *PGC-1α* is a master regulator of mitochondrial biogenesis and function. Exercise activates the *AMPK* and *PGC-1α* signaling pathways, leading to improved exercise performance [6,7,8].

The foregoing highlight the potential value of ginseng extract and L-carnitine as supplements for enhancing exercise performance. Both supplements improve exercise endurance, reduce muscle fatigue, and enhance recovery time. The mechanisms underlying these effects are complex and involve multiple factors, including increased energy production, reduced oxidative stress, and activation of key signaling pathways such as the *AMPK* and *PGC-1α* pathways. Additional studies are required to clarify the specific molecular pathways through which these supplements exert their effects and to identify the optimal regimen for maximizing performance gains [9,10].

This study aimed to explore the potential benefits of ginseng extract and L-carnitine supplementation on exercise endurance in male Wistar rats. By investigating the alterations in hepatic and muscular glycogen content, blood biochemistry, antioxidant enzyme activity, and gene expression, we aimed to uncover novel insights into the mechanisms by which these compounds enhance exercise performance.

## 2. Materials and Methods

### 2.1. Ginseng and L-Carnitine Extract Solution Preparation

Commercial powdered ginseng extract (Hi Balanz Company Limited, Bangkok, Thailand) was prepared using a water extraction method with a ginseng-to-water ratio of 1:10, and administered to the experimental animals at doses of 250 and 500 mg/kg. Commercial L-carnitine (Thai Food and Chemicals, Samut Prakan, Thailand) was used for animal experiments at the same doses. The chemical compositions of both commercial ginseng extract and L-carnitine are described in the certificate of analysis provided by the company. The physicochemical properties of the ginseng extract and L-carnitine were determined (Table 1).

### 2.2. Chemicals

A 95% (*v*/*v*) ethanol solution was prepared using analytical-grade ethanol (≥99.8% purity; Carlo Erba Reagents, Val-de-Reuil, France). A 5% (*w*/*v*) phenol solution was prepared by dissolving 50 g phenol (Sigma-Aldrich, St. Louis, MO, USA) in solvent. Potassium hydroxide and sodium sulfate solutions (30% *w*/*v*) were prepared by dissolving 180 g of potassium hydroxide (Sigma-Aldrich) in solvent. A stock glycogen standard solution was prepared by dissolving 50 mg of bovine liver glycogen powder (Sigma-Aldrich) in 10 mL of solvent to achieve a concentration of 5 mg/mL.

### 2.3. Experimental Designs

Male Wistar rats, weighing approximately 150–200 g and no older than six weeks, were obtained from the Animal Research Unit, Suranaree University of Technology. The rats were housed in a controlled environment with a constant temperature of 22 ± 3 °C and a 12 h light/dark cycle. Bedding was changed each day and the animals were provided standard rodent chow and water ad libitum. The experiment lasted 16 days. All experimental procedures were approved by the Institutional Animal Care and Use Committee of the Suranaree University of Technology (approval code 11/2017, approval date: 5 October 2016). Optimal rat housing typically involves a temperature range of approximately 22 ± 2 °C, relative humidity maintained between 45% and 65%, and an air exchange rate of 15–20 air changes per hour. A consistent 12 h light/dark cycle is crucial for well-being. As rats are sensitive to noise, maintaining noise levels below 85 dB is essential to minimize stress and ensure comfort. Rats were fed a commercial diet (CP). The manufacturer milled the feed according to the required specifications. Food was provided ad libitum through stainless-steel wirebar lid feeders. Chlorinated reverse osmosis water free of *Pseudomonas aeruginosa* was provided as drinking water (chlorine concentration 5–6 ppm). Water was provided in bottles containing sipper tubes. The CP brand is used for rodents (commercial diet) and was sterilized before use. The formulations of the nutritional diets are summarized in Table 2.

A 3 mL syringe was attached to an 18-gavage feeding tube (for rats) approximately 7 cm in length. One milliliter of the test substance was drawn into a syringe. Each rat was restrained by gently grasping the scruff of its neck with the left index finger and thumb. The remaining fingers were used to secure the body. This restraint method prevented the rat from struggling and caused its mouth to open slightly. The feeding tube was gently inserted over the tongue into the throat and advanced until an appropriate depth was reached. The substance was administered at a controlled rate to prevent esophageal injury or aspiration.

#### 2.3.1. Exercise Model

Fifty male Wistar rats, weighing approximately 200–250 g, were randomly assigned to ten experimental groups of five animals each. These groups included a control group, and the treatment group received ginseng extract and 2 different doses of L-carnitine at 250 mg/kg for the low-dose group and 500 mg/kg for the high-dose group, with or without an exercise regimen (6 days/week). The doses were calculated by converting the human dose used in normal cases to the rodent dose based on the following criteria:

Rat dose (mg/kg) = (human dose [mg/kg] × 70 kg × 0.018)/200

Supplement administration and posology also differed between groups. The European Union herbal monograph on *Panax ginseng* C.A. Mey, Radix, states that a single dose of powdered herbal substance should be 250–1200 mg (daily dose of 600–2000 mg) for adults and the elderly. When using extracts, a single dose depends on the method of preparation and can range from 40 to 360 mg (40 to 670 mg daily) for dry extracts. Different single and daily doses are listed for soft extracts (single dose: 219–440 mg, daily dose: 440–700 mg) and still others for liquid extracts (single dose: 500 mg–9.9 g; daily dose: 900 mg–19.8 g) [9].

The exercise protocol aimed to understand how exercise intensity and duration affect a specific outcome, although this outcome is not explicitly defined in the table. Following a weeklong acclimatization period without exercise, the length of exercise was gradually increased from 30 to 60 min over the next eight days. The 60 min daily exercise was maintained for the next four days, followed by a rest day. On day 15, the intensity returned to 60 min, followed by another rest day. The “exhaustive time (min)” metric, likely measured during exercise sessions, could represent the time taken to reach exhaustion or a predefined endpoint. Rats performed the swimming exercise six days per week. On the first day, rats swam for 30 min. Swimming duration was increased by 10 min each day until it reached 60 min. No exercise was performed on day 7. On day 16, muscle fatigue was induced by attaching a weight equivalent to 3% of the rat’s body weight to the tail and submerging the rat for 10 s. Uncoordinated movements and remaining in the water for 10 s without swimming at the surface were criteria for exhaustion. At this point, the rats were rescued and swimming time was recorded for each rat.

Following the experiment, the liver and muscle glycogen contents were measured using a modified version of a previously described method [10]. Blood samples for glucose, insulin, and free fatty acid levels were collected for biochemical analyses.

#### 2.3.2. Determination of Blood Biochemical Variables

The rats were euthanized by CO_2_ inhalation, followed by cervical dislocation. The rats were then placed in a supine position on a paraffin tray. A midline incision was made followed by rib removal to expose the thoracic cavity. The apex of the heart was carefully grasped with forceps, and an 18-gauge needle was inserted into the left ventricle to withdraw approximately 3–4 mL of blood. Blood was collected in a tube containing 2 mg EDTA and used for hematological and biochemical analyses. Blood samples (1 mL) were collected by cardiac puncture and allowed to clot at a temperature of approximately 15 °C for 15 min. The samples were then centrifuged at 1006.2× *g* for 5 min to separate serum from blood cells. The serum was carefully aspirated using a dropper and transferred to cleanly labeled eppendorf tubes. Serum samples were refrigerated at 4 °C for subsequent biochemical analyses.

#### 2.3.3. Determination of Organ Weight

Following the experimental protocol, animals were euthanized. The forelimbs and hindlimbs were dissected and the muscle tissues were carefully separated from connective tissues and weighed. Similarly, the liver was dissected, isolated from surrounding tissues and the gastrointestinal tract, and weighed after removing any adhering adipose tissue. All organ weights were recorded using a four-place analytical balance and expressed as relative weights (%g).

#### 2.3.4. Determination of Glycogen Levels in Tissue Samples

This study used a previously described method [11] to measure glycogen levels in the liver and gastrocnemius muscle samples. Briefly, 25 mg tissue samples were homogenized in 30% KOH saturated with Na_2_SO_4_, followed by precipitation with 95% ethanol. The precipitated glycogen was dissolved in double-distilled water and reacted with phenol-sulfuric acid. Absorbance was measured at 490 nm, and glycogen content was calculated using a standard curve. All assays were performed in triplicate.

#### 2.3.5. Gene Expression Analysis

Total RNA was isolated from 400 mg of liver and muscle tissue using a NucleoSpin RNA kit (Macherey-Nagel, Düren, Germany) according to the manufacturer’s protocol. cDNA was synthesized using the qPCR RT Master Mix (Vivantis Technologies, Selangor, Malaysia) following the manufacturer’s instructions. qPCR was performed to quantify the expression levels of glyceraldehyde 3-phosphate dehydrogenase (GAPDH), *AMPKα*1, *AMPKα*2, and *PGC-1α* using gene-specific primers [Table S1]. qPCR was performed using qPCRBIO SyGreen Mix Lo-ROX (PCR Biosystems, London, UK) on a model C1000Touch Thermal Cycler (BioRad Laboratories, Hercules, CA, USA). qPCR involved reverse transcription at 50 °C for 30 min, initial denaturation at 95 °C for 10 min, followed by 40 cycles of denaturation at 95 °C for 30 s, annealing at 56 °C (for *GAPDH*, *AMPKα1*, and *AMPKα2*) or 60 °C (for *PGC-1α*) for 1 min, and extension at 72 °C for 50 s.

#### 2.3.6. Malondialdehyde (MDA) Assay

This assay was based on the reaction between MDA and thiobarbituric acid (TBA) in an acidic environment, which forms a pink complex that absorbs light at 532 nm [12]. Trichloroacetic acid (TCA) reagent was prepared by dissolving 100 g of TCA in 100 mL of 0.6 M HCl. This reagent was stored at room temperature. PCR amplification was performed on the aforementioned C1000Touch Thermal Cycler by reverse transcription at 50 °C for 30 min, initial denaturation at 95 °C for 10 min, 40 cycles of denaturation at 95 °C for 30 s, annealing at 56 °C (for *GAPDH*, *AMPKα1*, and *AMPKα2*) or 60 °C (for *PGC-1α* and *GAPDH*) for 1 min, and extension at 72 °C for 50 s (Table 1). TBA solution (0.12 M) was prepared by dissolving 17.298 g TBA in 1000 mL of 0.26 M Tris. The solution was stored at room temperature and filtered prior to use. A 10 mM stock solution of MDA was prepared by adding 20.8 µL of tetramethoxypropane to HCl and diluting to 10 mL with distilled water. A 100 µM working standard was prepared by diluting the stock solution, and a standard curve was generated using serial dilutions of this standard.

### 2.4. Statistical Analyses

Data are presented as the mean ± either the standard error of the mean (SEM) or standard deviation, depending on the experiment. Statistical analyses were performed using an analysis of variance (ANOVA) in SPSS version 16.0 (SPSS Inc., Chicago, IL, USA). Animals that died during the experiment were excluded from statistical analysis. A Tukey–Kramer post hoc test was performed, with *p* < 0.05 indicating statistical significance, to compare the differences between the experimental and control groups. Pathological changes and mortality rates were descriptively analyzed and compared with those in the control group.

### 2.5. Experimental Procedures and Data Collection

All experiments were conducted in the Chemistry Laboratory, Instrument Building 3, and the Animal Facility in Instrument Building 14. Both are located within the Instrumental Analysis Unit of the Science and Technology Instrumentation Center at Suranaree University of Technology.

## 3. Results

### 3.1. Effects on Exercise Endurance

**Figure 1:**
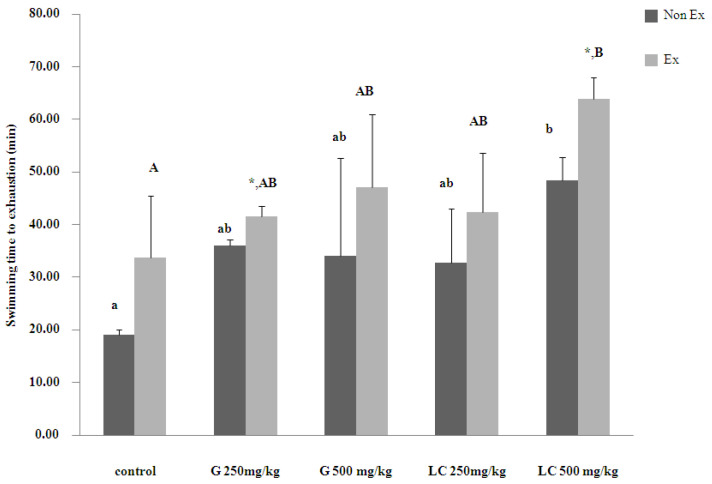
Impact of 16 days of supplementation with ginseng extract (G) or L-carnitine (LC) on exercise endurance in male Wistar rats. Data are presented as mean ± SEM (n = 5 per group). Asterisks (*) denote significant differences between non-exercise (Non-Ex) and exercise (Ex) groups receiving same treatment. Lowercase and uppercase letters indicate significant differences within Non-Ex and Ex group, respectively. Means sharing a common superscript do not differ significantly (*p* > 0.05).

L-carnitine (500 mg/kg) significantly improved exercise endurance in both sedentary and trained male rats (Figure 1). In sedentary rats, this concentration of L-carnitine extended swimming time to 48 min. Among the trained rats, 500 mg/kg L-carnitine supplementation yielded the longest post-exhaustion swimming time of 63 min, followed by 500 mg/kg ginseng extract (47 min), 250 mg/kg L-carnitine (42 min), and 250 mg/kg ginseng extract (41 min). Supplementation with both L-carnitine and ginseng extracts enhanced exercise endurance compared to the control group.

### 3.2. Effects on Average Daily Weight Gain

**Figure 2:**
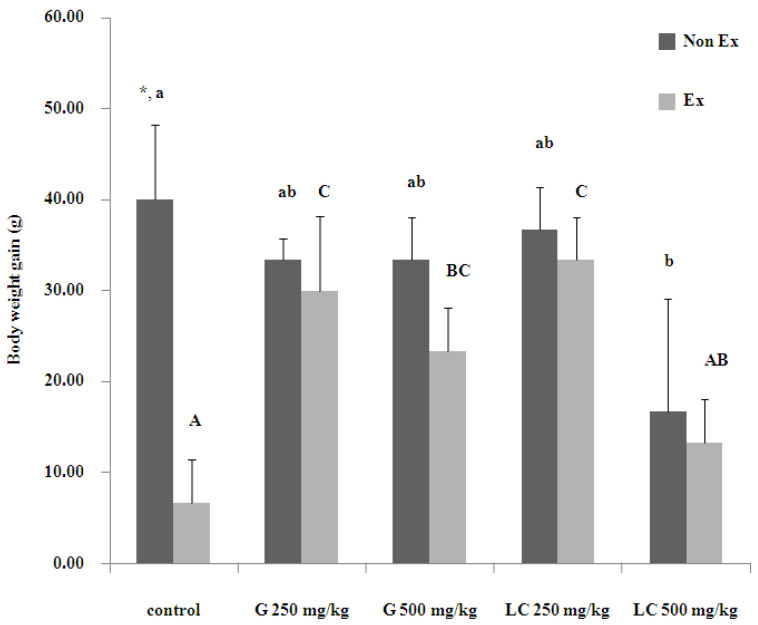
Effects of the 16-day administration of ginseng extract (G) and L-carnitine (LC) on body weight gain. Data are presented as the mean ± standard error of the mean (SEM); n = 5 per group. * Denotes a significant difference between non-exercised (Non-Ex) and exercised (Ex) groups receiving the same treatment. Lowercase and uppercase letters indicate significant differences within the Non-Ex and Ex group, respectively. Means sharing the same superscript are not significantly different from each other (*p* < 0.05, two-way ANOVA with Duncan’s multiple range test).

Figure 2 shows the average daily weight gain of the rats. Sedentary rats administered ginseng or L-carnitine tended to gain less weight than controls. This effect was especially evident for L-carnitine. In contrast, exercised rats supplemented with 250 mg/kg ginseng or L-carnitine gained significantly more weight than rats in the other groups. Exercise alone increased weight gain compared to sedentary controls. These results suggest that the effects of ginseng and L-carnitine on weight gain may differ depending on the exercise status.

### 3.3. Relative Organ Weight

As shown in Table 3, the relative weight of the examined organs remained unchanged across the exercise and sedentary groups. The findings indicate that supplementation with ginseng extract and L-carnitine combined with the exercise regimen did not induce any observable changes in the size of these organs.

### 3.4. Blood Chemical Parameters

Table 4 shows blood biochemistry results. No significant differences were found in the levels of glucose, triglycerides, aspartate aminotransferase (AST), alanine aminotransferase (ALT), blood urea nitrogen (BUN), and insulin. However, sedentary rats receiving 500 mg/kg L-carnitine had significantly higher creatinine levels. Creatinine and BUN are waste products primarily excreted by the kidneys. Elevated creatinine may suggest potential kidney function impairment in the sedentary group, although further investigation is needed. Insulin regulates carbohydrate metabolism and influences lipid and protein metabolism. AST, ALT, and LDH are enzymes that can indicate tissue damage when elevated in the blood.

### 3.5. Glycogen Contents in Liver and Muscle

**Figure 3:**
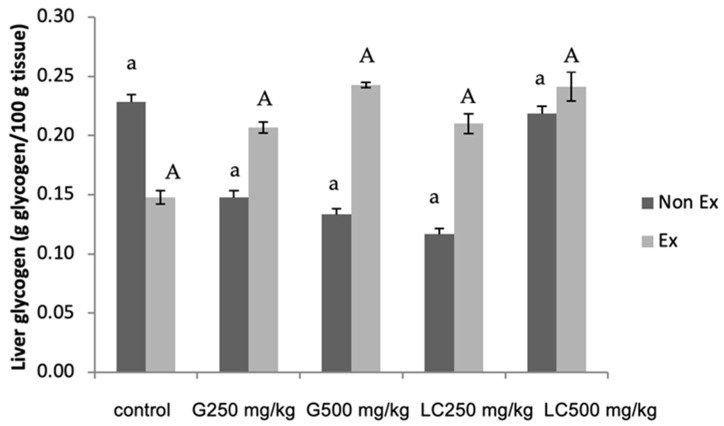
Effects of the 16-day administration of ginseng extract (G) and L-carnitine (LC) on hepatic glycogen content. Values are expressed as mean ± SEM (n = 5 per group). Small letters indicate significant differences within the Non-Ex group. Capital letters indicate significant differences within the Ex-group. Means sharing the same superscript are not significantly different from each other (*p* < 0.05, two-way ANOVA with Duncan’s multiple range test).

The hepatic glycogen content was significantly increased in all groups supplemented with either 250 or 500 mg/kg ginseng extract or L-carnitine, regardless of exercise training, compared with the control group (Figure 3). Sedentary controls displayed lower glycogen levels than exercise controls. Hepatic glycogen is crucial for exercise performance, as it fuels energy production. Elevated glucose metabolism decreases hepatic glycogen levels; increased muscle glycogen levels can lead to fatigue during prolonged exercise [13]. Consuming a high-carbohydrate diet can improve exercise endurance by increasing glycogen stores in both the liver and muscles [14,15].

### 3.6. Muscle Glycogen Content

**Figure 4:**
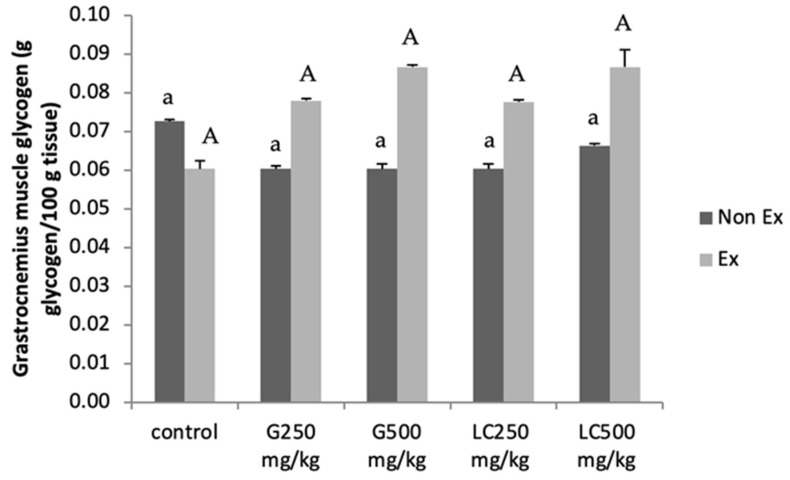
Effects of the 16-day intervention with ginseng extract (G) and L-carnitine (LC) on skeletal muscle glycogen content were examined. Data are presented as the mean ± standard error of the mean (SEM); n = 5 per group. Lowercase letters indicate significant differences within the Non-Ex group. Uppercase letters indicate significant differences within the Ex-group. Means sharing the same superscript are not significantly different from each other (*p* < 0.05, two-way ANOVA; Duncan’s multiple range test).

As shown in Figure 4, muscle glycogen content was significantly higher in rats supplemented with ginseng extract and L-carnitine, regardless of exercise training, than that in the control group. The increased muscle glycogen content can be attributed to pre-exercise consumption of these supplements, which enhances exercise endurance.

### 3.7. Effects of Ginseng Extract and L-Carnitine on Antioxidant Enzymes

Ginseng extract significantly increased the expression of genes encoding superoxide dismutase (SOD) and catalase, leading to enhanced antioxidant enzyme activity in the liver and skeletal muscles (Table 5). The findings suggest the potential involvement of mechanisms such as the activation of antioxidant response elements or upregulation of antioxidant gene expression. By modulating the cellular redox environment, ginseng extract and L-carnitine may contribute to improved health outcomes by reducing oxidative stress and enhancing cellular defense mechanisms. Supplementation with ginseng extract slightly increased SOD activity and decreased the levels of MDA and reactive oxygen species (ROS) in both the exercise and sedentary groups. Both ginseng extract and L-carnitine reduced ROS levels, especially in the exercise group. Antioxidants are crucial for preventing muscle fatigue by mitigating oxidative stress [4]. By reducing oxidative stress, ginseng extract and L-carnitine may help protect cells and enhance exercise tolerance [5].

### 3.8. Effects of Ginseng Extract and L-Carnitine on Gene Expression in Liver and Muscle

#### 3.8.1. Expression of *AMPKα1* Gene

**Figure 5:**
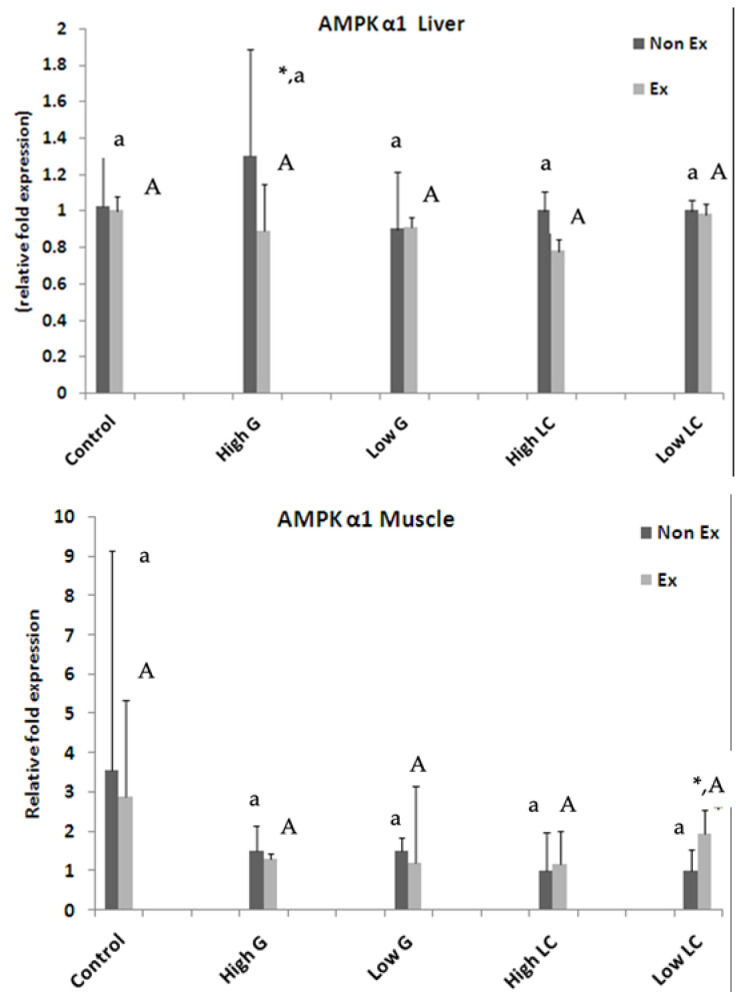
Following a 2-week treatment period with ginseng extract (G) and L-carnitine (LC), subjects underwent an exhaustive swimming exercise. To assess the relative expression of the AMPK α1 gene, mRNA levels in the liver (**top** panel) and muscle tissue (**bottom** panel) were quantified using semi-quantitative RT-PCR. Data are presented as the mean ± standard error of the mean (SEM) (n = 3). * indicates a significant difference between non-exercise training (Non-Ex) and exercise training (Ex) groups with the same treatment. Lowercase letters denote significant differences within the Non-Ex group, while uppercase letters indicate significant differences within the Ex group. Means sharing the same superscript are not significantly different from each other (*p* < 0.05, two-way ANOVA; Duncan’s multiple comparison test).

Figure 5 shows *AMPK α1* gene expression in the liver (upper panel) and muscle (lower panel). Sedentary rats supplemented with 500 mg/kg ginseng extract displayed significantly increased *AMPK α1* expression in the liver compared to all other groups. Exercise reduced *AMPK α1* expression in the ginseng- and L-carnitine-supplemented group. In muscle, L-carnitine supplementation tended to increase *AMPK α1* expression in sedentary rats, and 250 mg/kg L-carnitine significantly increased the level in exercised rats. Previous studies have shown that exercise increases *AMPK* phosphorylation, which is triggered by decreased glycogen levels. This activation promoted energy balance and glucose uptake. In rodent models, increased *AMPK* phosphorylation correlates with decreased muscle glycogen content [8].

#### 3.8.2. *AMPK α2* Gene Expression

**Figure 6:**
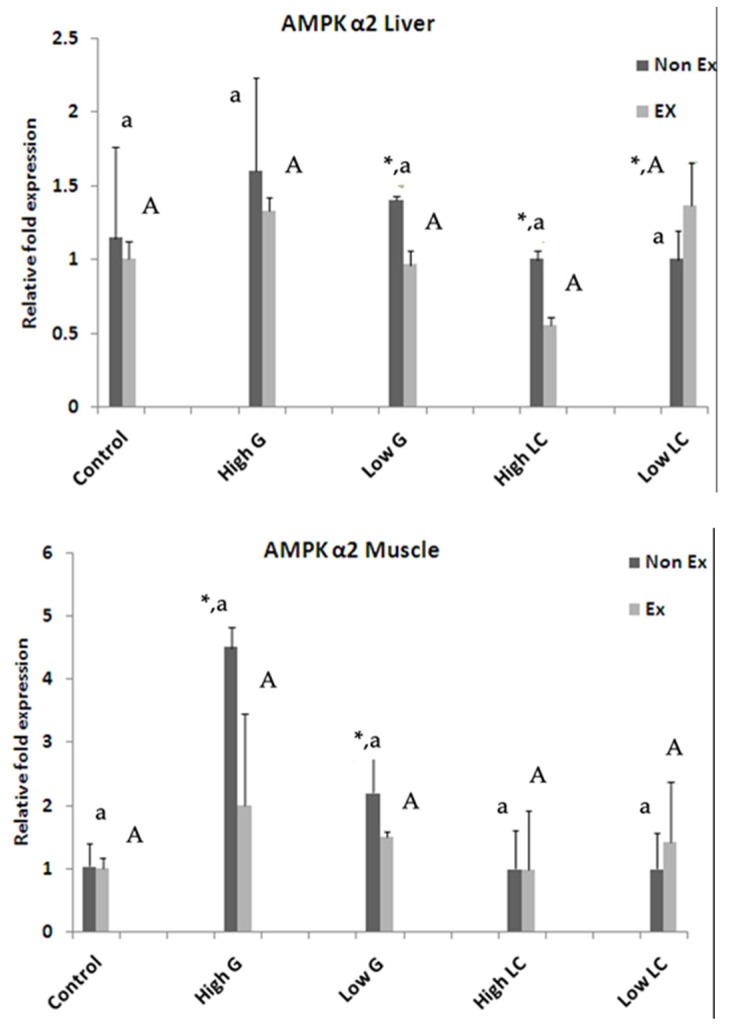
Following a two-week supplementation period with ginseng extract (G) and L-carnitine (LC), mice underwent an exhaustive swimming test. To assess the relative expression of AMP-activated protein kinase α2 (*AMPK α2*), semi-quantitative RT-PCR was employed to quantify mRNA levels in both liver and muscle tissues post-exercise. Data are presented as the mean ± SEM (n = 3). Significant differences between non-exercised (Non-Ex) and exercised (Ex) groups within each treatment are indicated by asterisks (*). Small letters denote significant differences within the Non-Ex group, while capital letters indicate significant differences within the Ex-group. Means sharing the same superscript are not significantly different (*p* < 0.05, two-way ANOVA with Duncan’s multiple comparison test).

The upper panel in Figure 6 presents the expression of the *AMPK α2* gene in the liver. In the sedentary group, rats supplemented with ginseng extract exhibited significantly higher *AMPK α2* gene expression compared to all other groups. However, in the exercise group, rats supplemented with 250 mg/kg ginseng extract and 500 mg/kg L-carnitine showed higher *AMPK α2* gene expression compared to other groups. When comparing the exercise and sedentary groups, no significant difference was observed in *AMPK α2* gene expression in the exercise group. The lower panel of Figure 6 shows the expression of the *AMPK α2* gene in muscle. In the sedentary group, rats supplemented with ginseng extract exhibited significantly higher *AMPK α2* gene expression compared to all other groups. No significant differences were observed in *AMPK α2* gene expression among the exercise groups. However, *AMPK α2* gene expression was lower in the exercise groups compared to the sedentary groups.

#### 3.8.3. Expression of PGC-1α Gene

**Figure 7:**
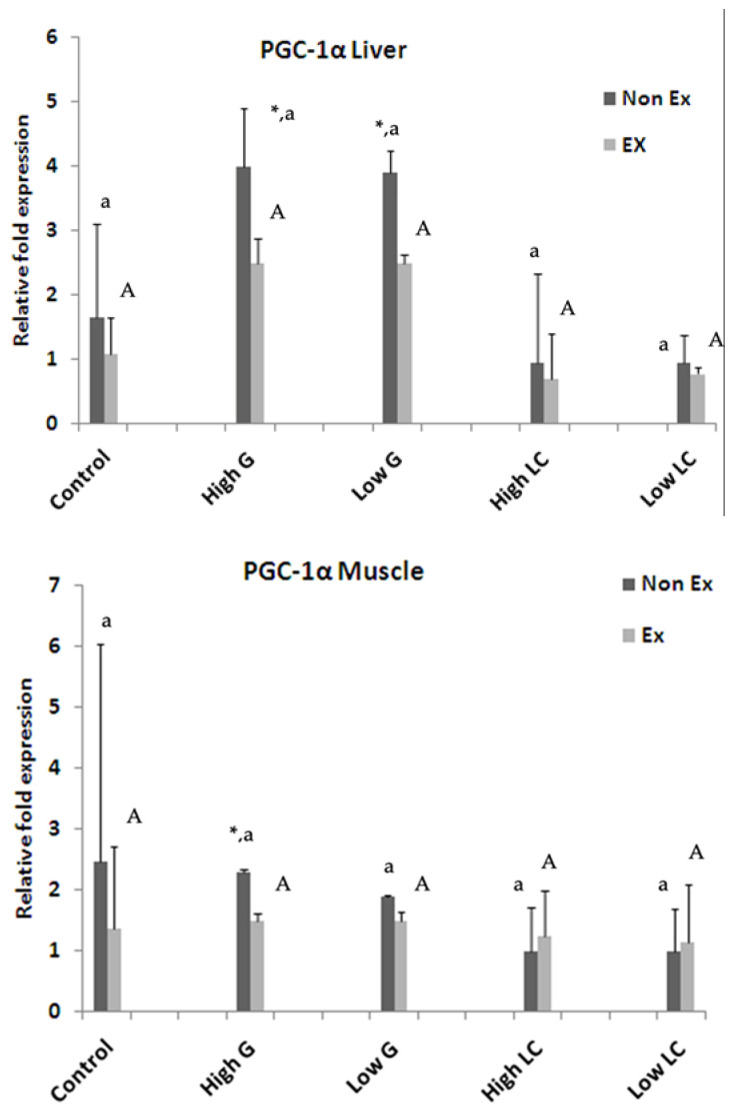
After a 2-week supplementation period with ginseng extract (G) and L-carnitine (LC), mice underwent exhaustive swimming. To assess the impact on mitochondrial biogenesis, the mRNA expression of peroxisome proliferator-activated receptor gamma coactivator 1-alpha (PGC-1α) was measured in liver and muscle tissues using semi-quantitative RT-PCR. Data are presented as the mean ± SEM (n = 3). Significant differences between non-exercised (Non-Ex) and exercised (Ex) groups within each treatment are indicated by *. Means sharing the same superscript were not significantly different (*p* < 0.05, two-way ANOVA with Duncan’s post hoc test).

Ginseng supplementation significantly impacted hepatic *PGC-1α* gene expression in sedentary rats (Figure 7). Exercise groups receiving ginseng showed no significant differences in *PGC-1α* gene expression. Muscle *PGC-1α* gene expression was significantly different (*p* < 0.05) among the 500 mg/kg ginseng-supplemented groups (Figure 7). In the 500 mg/kg ginseng group, exercise suppressed *PGC-1α* gene expression compared to sedentary controls. Exercise strongly induces *PGC-1α* expression [7].

## 4. Discussion

Supplementation with ginseng extract and L-carnitine supplementation significantly enhanced exercise endurance in male Wistar rats, particularly in the exercise group. These findings are consistent with those of previous studies that highlighted the ergogenic effects of these compounds [1,2,3].

Numerous studies have confirmed that the ergogenic potential of ginseng can enhance endurance, reduce fatigue, and improve recovery time [1,2]. These effects are likely mediated by increased energy production, reduced oxidative stress, and improved muscle function. These findings support the observations, showing that ginseng increased liver glycogen content and upregulated key metabolic genes like AMPK and PGC-1α [14,15]. L-Carnitine, a crucial player in fatty acid oxidation, transports long-chain fatty acids into the mitochondria, facilitating energy production and contributing to the observed enhancement of exercise endurance [3]. Furthermore, the combination of ginseng and L-carnitine may exhibit synergistic effects. Ginseng may enhance the effects of L-carnitine by increasing glycogen availability and reducing oxidative stress [1,2], while L-carnitine may spare glycogen by improving fatty acid utilization [3].

Regarding physiological parameters, L-carnitine (500 mg/kg) increased body weight in sedentary rats, whereas ginseng (250 mg/kg) showed the most significant weight gain in exercised rats, suggesting the potential anabolic effects of ginseng [1] and the more complex influence of L-carnitine on body weight [3]. No significant alterations were observed in the relative weights of major organs, including the liver, soleus, extensor digitorum longus (EDL), and gastrocnemius muscles. Blood biochemistry analyses revealed that the supplements had no significant effects on blood glucose or insulin levels. While liver enzymes remained within normal limits, a significant increase in creatinine levels was observed in the sedentary group receiving 500 mg/kg L-carnitine, potentially indicating an impact on kidney function that requires further investigation. The significant increase in creatinine levels observed in the sedentary group administered 500 mg/kg L-carnitine warrants further investigation. Potential mechanisms include renal clearance overload because of high L-carnitine doses, particularly in sedentary animals with potentially reduced renal function. Indirect effects such as disruptions to the electrolyte and fluid balance may also contribute. Individual variability in L-carnitine metabolism and potential susceptibility to adverse effects cannot be ruled out. Further research is necessary, including histopathological examinations, the assessment of other kidney function markers, investigation of dose-dependent effects, and exploration of the potential mitigating effects of exercise on L-carnitine-induced renal changes [16,17].

Triglyceride levels were unaffected. Importantly, both supplements significantly increased the liver glycogen content, particularly in the exercise group [18,19,20], whereas no significant changes were observed in the gastrocnemius muscle glycogen content. Males and females exhibit physiological and metabolic differences. These differences can significantly influence the response of the body to various interventions, including supplementation. For example, hormonal variations between the sexes can affect insulin sensitivity, glucose metabolism, and fat oxidation. The findings of this study may not be directly applicable to female rats or other animals. Extrapolating the results to females or humans should be performed with caution as the observed effects may not be consistent across different sexes and species.

Intense exercise generates ROS, causing oxidative stress that damages the muscle tissue and impairs performance. Antioxidant supplementation may benefit endurance athletes by neutralizing ROS, potentially reducing muscle damage and inflammation, and improving recovery. This could lead to enhanced training capacity and improved performance. However, the effectiveness of antioxidant supplementation in endurance athletes remains uncertain, with some studies showing positive effects, whereas others do not. Further research is needed to determine the optimal type, dosage, and timing of antioxidant supplementation to achieve optimal benefits [21]. The book chapter by Nieman and Johannsen [22] highlighted the importance of vitamins and minerals, especially antioxidants, in supporting endurance sports performance. Antioxidants help protect the body from the oxidative stress caused by exercise, which can damage cells and impair performance. By neutralizing harmful free radicals, antioxidants can reduce muscle damage and inflammation and improve recovery, ultimately enhancing athletes’ ability to train harder and perform better [22].

During endurance training, AMPK activation stimulates key metabolic processes such as glucose uptake, fatty acid oxidation, and mitochondrial biogenesis. Importantly, AMPK also potently activates *PGC-1α*, a key regulator of mitochondrial biogenesis. This synergistic relationship between AMPK and *PGC-1α* enhances cellular energy metabolism, ultimately contributing to improved endurance performance [21]. Both ginseng and L-carnitine exhibit antioxidant properties, significantly reducing MDA levels and increasing SOD activity [18,19], potentially through mechanisms involving free radical scavenging and enhanced antioxidant enzyme activity. Furthermore, both supplements significantly increased *AMPK α-1* gene expression in the muscle, particularly in the exercise group [23,24]. AMPK activation plays a crucial role in energy metabolism, promoting glucose uptake, fatty acid oxidation, and mitochondrial biogenesis [7,8,13].

It is well-established that males and females exhibit physiological and metabolic differences. These differences can significantly influence how the body responds to various interventions, including supplementation. For example, hormonal variations between sexes can impact insulin sensitivity, glucose metabolism, and fat oxidation. Additionally, age and baseline fitness significantly influenced the study outcomes. Older rats may exhibit altered metabolic responses and muscle function compared to younger rats, potentially affecting their reactions to exercise and supplementation. Similarly, rats with varying baseline fitness levels may exhibit different responses. Highly trained rats may have pre-existing metabolic adaptations, potentially diminishing the observed effects of supplementation compared to sedentary rats. Furthermore, the chosen dosages of ginseng and L-carnitine may not be optimal for observing exercise-enhancing effects because the optimal dosages in animal models can vary considerably. Furthermore, the 16-day supplementation period might be insufficient to fully observe the effects of these supplements, as longer durations may be necessary to observe significant changes in exercise performance and the underlying metabolic adaptations.

Future studies should include a broader spectrum of study designs to elucidate the ergogenic potential of ginseng. This necessitates investigating a wider range of ginseng dosages to establish optimal dose–response relationships and identify potential dose-dependent effects on exercise performance and key metabolic parameters [1,2,17,18]. Furthermore, exploring the potential synergistic or antagonistic interactions of ginseng with other commonly used ergogenic aids such as creatine and caffeine is crucial for optimizing supplementation strategies for athletes. Finally, investigating the effects of ginseng across diverse exercise modalities, including endurance, strength, and high-intensity interval training, will provide valuable insights into its potential impact on various aspects of athletic performance.

In addition, the NOODLE study published in [25] investigated the accuracy of the recalibrated FRIEND equation in predicting peak oxygen pulse (O2Ppeak) in endurance athletes. The recalibrated equation provided more accurate predictions of O2Ppeak than the original equation, particularly in endurance athletes. This finding has significant implications for understanding the cardiovascular physiology of endurance athletes because O2Ppeak is a key indicator of cardiorespiratory fitness. The results of this study suggest that the recalibrated FRIEND equation may be a valuable tool for assessing and monitoring training progress in endurance athletes [25].

The present study involved male Wistar rats. The short-term design, focus on swimming endurance, and lack of a placebo group may limit the generalizability of our results to humans. Further research with longer duration, diverse exercise types, and human subjects is crucial to fully understand the long-term effects and underlying mechanisms of these supplements on exercise performance.

## 5. Conclusions

This study investigated the effects of ginseng extract and L-carnitine supplementation on various physiological parameters in male Wistar rats, particularly focusing on exercise endurance, body weight, organ weight, blood biochemistry, glycogen content, antioxidant enzymes, and gene expression. Both ginseng extract and L-carnitine significantly enhanced exercise endurance, likely owing to increased glucose levels and hepatic glycogenolysis. Although high doses of L-carnitine in sedentary rats may have negative effects on kidney function, no significant adverse effects were observed on other blood parameters. Both supplements increased the hepatic glycogen content, suggesting improved exercise performance. Additionally, both ginseng extract and L-carnitine exhibited antioxidant properties by reducing oxidative stress and increasing SOD activity. The upregulation of AMPK and PGC-1α gene expression by both supplements suggests their potential roles in enhancing energy metabolism and mitochondrial biogenesis. Overall, these results indicate that these supplements, particularly in combination with exercise, may offer potential benefits for exercise performance and metabolic health. However, further studies are required to elucidate the underlying mechanisms and the long-term effects.

## Figures and Tables

**Table 1:** Properties of ginseng extract and L-carnitine.

Components 1	Ginseng Extract	L-Carnitine
Appearance	Light brown powder	White crystalline powder
Bulk density	0.40–0.60 g/cc	0.5–0.55 g/mL
Active ingredient	Ginsenoside > 2%	L-carnitine 68.2 ± 1% L-tartaric acid 31.8 ± 1% D-carnitine ≤ 0.35%
Moisture %	5.89 ± 0.09	4.25 ± 0.02
Water solubility index (WSI) (%)	97.65 ± 0.25	98.90 ± 0.28

**Table 2:** Formulations of the experimental diets.

Ingredients ^a^	Control
Casein	140
DL-Methionine	2
Corn starch	435
Maltodextrin	150
Sucrose	100
Cassava DF	-
Cellulose	-
Soybean oil	50
Coconut oil	35
Mineral mix S10001	35
Calcium carbonate	5.5
Sodium chloride	8
Potassium citrate	10
Vitamin mix V10001	10
Choline bitartrate	2
Cholesterol	12.5
Sodium cholic acid	5
FD&C blue dye #1	0.1
FD&C Yellow dye #5	-
FD&C red dye #40	-

^a^ The ingredients are expressed as g/kg of diet (dry weight).

**Table 3:** Effects of the 16-day supplementation with ginseng extract (G) and L-carnitine (LC) on the relative weight of liver, soleus, extensor digitorum longus (EDL), and gastrocnemius muscles.

Group	Relative Organ Weight (g/100 g Body Weight)			
	Liver	Soleous	EDL	Grastrocnomius
Control				
Non-Ex	2.71 ± 0.03	0.06 ± 0.01	0.07 ± 0.01	1.10 ± 0.03
Ex	2.97 ± 0.11	0.07 ± 0.01	0.08 ± 0.03	1.13 ± 0.04
250 mg/kg G				
Non-Ex	2.97 ± 0.05	0.04 ± 0.00	0.04 ± 0.00	1.12 ± 0.01
Ex	2.87 ± 0.09	0.37 ± 0.00	0.04 ± 0.00	1.12 ± 0.01
500 mg/kg G				
Non-Ex	2.74 ± 0.16	0.04 ± 0.01	0.05 ± 0.01	1.13 ± 0.03
Ex	2.91 ± 0.15	0.04 ± 0.00	0.05 ± 0.00	1.13 ± 0.03
250 mg/kg LC				
Non-Ex	2.87 ± 0.13	0.04 ± 0.01	0.04 ± 0.00	1.13 ± 0.04
Ex	2.71 ± 0.13	0.04 ± 0.00	0.05 ± 0.01	1.13 ± 0.02
500 mg/kg LC				
Non-Ex	3.23 ± 0.23	0.07 ± 0.02	0.08 ± 0.03	1.13 ± 0.05
Ex	2.93 ± 0.26	0.06 ± 0.02	0.07 ± 0.03	1.13 ± 0.03

Values are expressed as mean ± SEM. Abbreviations: G = ginseng extract; LC = L-carnitine; Ex, exercise training; Non-Ex, non-exercise training.

**Table 4:** Effects of the 16-day administration of ginseng extract (G) and L-carnitine (LC) on blood biochemical parameters of male Wistar rats.

Group	Parameters							
	Glucose (mg/dL)	BUN (mg/dL)	Creatinine (mg/dL)	TG (mg/dL)	AST (U/L)	ALT (U/L)	LDH (U/L)	Insulin (µIU/L)
Control								
Non-Ex	94.33 ± 27.42	19.46 ± 1.20	0.36 ± 0.01 (b)	81.66 ± 9.86	183 ± 58.20	31 ± 1.00	2410 ± 769.99	2 ± 0.00
Ex	100 ± 8.88	22.96 ± 1.25	0.51 ± 0.09	142 ± 63.37	163.66 ± 58.15	40.66 ± 6.02	6424 ± 1733.56 (*)	2 ± 0.00
250 mg/kg G								
Non-Ex	93.6 ± 17.78	16.4 ± 3.05	0.57 ± 0.32 (b)	106 ± 10.81	444 ± 248.81	44.66 ± 14.64	8592 ± 2766.19	2 ± 0.00
Ex	101.33 ± 30.13	12.46 ± 5.82	0.52 ± 0.12	64 ± 20.66	256 ± 112.46	50 ± 17.43	4374 ± 643.54	2 ± 0.00
500 mg/kg G								
Non-Ex	69.66 ± 3.78	25.4 ± 5.74	0.4 ± 0.09 (b)	120.33 ± 17.95	221.66 ± 38.81	35.66 ± 5.5	4336 ± 1767.02	2 ± 0.00
Ex	112 ± 84.33	19.46 ± 4.64	0.31 ± 0.02	103.66 ± 17.61	414.33 ± 393.78	51 ± 23.64	4921.33 ± 2283.56	4.0 ± 3.50
250 mg/kg LC								
Non-Ex	85 ± 7.93	20.83 ± 3.30	0.38 ± 0.10 (b)	123.33 ± 23.45	278.33 ± 107.54	39 ± 7.81	7603.66 ± 3693.31	2 ± 0.00
Ex	76 ± 24.97	27.76 ± 11.56	0.48 ± 0.11	98.66 ± 21.12	208.66 ± 31.81	34 ± 8.71	7915 ± 49.79	2 ± 0.00
500 mg/kg LC								
Non-Ex	92 ± 5.65	19.45 ± 3.46	1.05 ± 0.0 (a)	129 ± 29.69	202.5 ± 194.45	30.5 ± 3.53	3752.5 ± 757.31	2 ± 0.00
Ex	83.33 ± 9.71	24.5 ± 1.64	0.62 ± 0.37	116.66 ± 28.36	131 ± 11.26	39 ± 10.44	6023.66 ± 3746.4	2 ± 0.00

Values are expressed as the mean ± SEM (n = 5 per group). * Indicates a significant difference between the Non-Ex and Ex-groups following the same treatment. Small letters indicate significant differences between the non-Ex and Ex groups. Capital letters indicate significant differences between the Ex-groups. Abbreviations: G = ginseng extract; LC = L-carnitine; Ex, exercise training; Non-Ex, non-exercise training; TG, triglyceride; BUN, blood urea nitrogen; AST, aspartate aminotransferase; ALT, alanine aminotransferase; LDH, lactate dehydrogenase.

**Table 5:** Effects of ginseng extract and L-carnitine on antioxidant enzymes in swimming rats.

Groups		Parameter		
		MDA (mmol/mg)	SOD (U/mg)	ROS (FI/g)
Control	Non-Ex	12.2 ± 0.9 (b)	270.5 ± 17.8 (a)	1516.5 ± 59.9 (c)
	Ex	11.8 ± 0.5 (B)	240.9 ± 19.8 (A)	1606.7 ± 80.0 (B)
250 mg/kg Ginseng extract	Non-Ex	11.0 ± 0.2 (ab)	269.6 ± 16.9 (a)	1456.8 ± 34.5 (b)
	Ex	10.7 ± 0.5 (AB)	275.9 ± 12.4 (AB)	1400.5 ± 23.2 (AB)
500 mg/kg Ginseng extract	Non-Ex	10.9 ± 0.5 (ab)	265.4 ± 14.7 (a)	1378.5 ± 53.8 (a)
	Ex	10.3 ± 0.5 (A)	280.8 ± 11.5 (AB)	1350.9 ± 34.6 (A)
250 mg/kg L-carnitine	Non-Ex	10.7 ± 0.3 (ab)	298.4 ± 15.1 (b)	1465.6 ± 45.2 (b)
	Ex	9.97 ± 0.9 (A)	320.9 ± 23.5 (B)	1320.3 ± 34.8 (AB)
500 mg/kg L-carnitine	Non-Ex	10.3 ± 0.5 (a)	296.5 ± 16.9 (b)	1398.1 ± 45.8 (a)
	Ex	8.89 ± 0.9 (A)	350.8 ± 17.6 (B)	1250.6 ± 25.8 (A)

Data are presented as the mean ± standard error of the mean (SEM); n = 5 per group. Lowercase letters indicate significant differences within the Non-Ex group and uppercase letters indicate significant differences within the Ex-group. Means sharing the same superscript are not significantly different (*p* < 0.05, two-way ANOVA with Duncan’s multiple comparison test).

## Data Availability

The original contributions presented in the study are included in the article, and further inquiries can be directed to the corresponding authors.

## Supplementary Materials

The following supporting information can be downloaded at https://www.mdpi.com/article/10.3390/nu17030568/s1, Table S1: Primer sequences.
