# Supplementary material for: Role of Ginseng and L-Carnitine in Modulating Exercise Endurance and Oxidative Stress in Rats
Source: Nutrients. 2025 Feb 3;17(3):568. doi: 10.3390/nu17030568 (PMC11819988; doi:10.3390/nu17030568)
Supplement: Supplementary file 1 [file nutrients-17-00568-s001.zip › nutrients-3420259-supplementary.pdf]

Table S1. Primer sequences

Gene name	Primer	Primer Sequence (5' to 3')
AMPK α1	Reverse	GGG AGG TCA CGG ATC AGG
	Forward	GGG ATC CAT CAG CAA CTA TCG
AMPK α2	Reverse	TGT CGT ATG GTT TGC TCT GG
	Forward	TCG CAG TGG CTT ATC ATC TC
PGC-1α	Reverse	GGCCTGCAGTTCCAGAGAGT
	Forward	GACCCCAGAGTCACCAAATGA
GAPDH	Reverse	GGC ATG GAC TGT GGT CAT GAG
	Forward	TGC ACC ACC AAC TGC TTA GC
